# Expression of NLRP3 inflammasome in leprosy indicates immune evasion of *Mycobacterium leprae*

**DOI:** 10.1590/0074-02760190324

**Published:** 2020-02-27

**Authors:** Ana Luisa Gomes Mendes, Heloísa Di Matteo Joaquim, Mara Inês Stefanini Zamae, Ramon Meira Assis, Jéssica Renata de Moura Peixoto, Margarida Maria Gomes de Araújo, Antônio Carlos Martins Guedes, Edward José Oliveira, Vanessa Peruhype Magalhães, Marcelo Antônio Pascoal-Xavier

**Affiliations:** 1 Universidade Federal de Minas Gerais, Faculdade de Medicina, Departamento de Anatomia Patológica e Medicina Legal, Belo Horizonte, MG, Brasil; 2 Universidade Federal de Minas Gerais, Faculdade de Medicina, Departamento de Clínica Médica, Belo Horizonte, MG, Brasil; 3 Fundação Oswaldo Cruz-Fiocruz, Instituto René Rachou, Belo Horizonte, MG, Brasil

**Keywords:** leprosy, inflammasome, NLRP3, caspase

## Abstract

**BACKGROUND:**

Leprosy is an infectious-contagious disease caused by *Mycobacterium leprae* that remain endemic in 105 countries. This neglected disease has a wide range of clinical and histopathological manifestations that are related to the host inflammatory and immune responses. More recently, the inflammasome has assumed a relevant role in the inflammatory response against microbiological agents. However, the involvement of inflammasome in leprosy remains poorly understood.

**OBJECTIVES:**

The aim is to associate biomarkers of inflammasome with the different immunopathological forms of leprosy.

**METHODS:**

We performed an observational, cross-sectional, and comparative study of the immunophenotypic expression of inflammasome-associated proteins in immunopathological forms of leprosy of 99 skin lesion samples by immunohistochemistry. The intensity and percentage of NLRP3, Caspase-1, Caspases-4/5, interleukin-1β and interleukin-18 immunoreactivities in the inflammatory infiltrate of skin biopsies were evaluated.

**FINDINGS:**

Strong expression of NLRP3 and inflammatory Caspases-4/5 were observed in lepromatous leprosy (lepromatous pole). In addition, were observed low expression of caspase-1, interleukin-1β, and interleukin-18 in tuberculoid and lepromatous leprosy. The interpolar or borderline form showed immunophenotype predominantly similar to the lepromatous pole.

**MAIN CONCLUSIONS:**

Our results demonstrate that the NLRP3 inflammasome is inactive in leprosy, suggesting immune evasion of *M. leprae*.

Leprosy is a chronic, neglected infectious-contagious disease caused by *Mycobacterium leprae*, an obligate, intracellular, alcohol-acidic bacillus. Despite the sustained reduction in its global prevalence, at 0.32 per 10,000 population by 2014, a total of 105 countries remain endemic or with one or more patients for every 10,000 inhabitants.^1^

The leprosy spectrum presentation in the clinical and immunopathological context has led Ridley and Jopling to recognise five forms of the condition: polar tuberculoid (TT), lepromatous leprosy (LL), intermediate borderline tuberculoid (BT), borderline borderline (BB), and borderline lepromatous (BL). A sixth form, indeterminate leprosy (IL), is also commonly used.^2^

Previous studies indicated a close relationship between the immunity status of the host and leprosy spectrum.^3^ ^,^ ^4^ ^,^ ^5^ Some recognise the adaptive immunity as responsible for tissue destruction.^6^ However, recent data suggest that the innate immune response is also critical in defining the course of *M. leprae* infection and, ultimately, the clinical outcome, including tissue destruction.^7^ Accordingly, the complement system, as the first line of defense against pathogens and a key component of innate immunity, has been shown to modulate the adaptive immune response and cause leprosy related to nerve damage.^8^ ^,^ ^9^ *M. leprae* is initially recognised by various innate immune receptors, including Toll-like receptors.^10^ ^,^ ^11^ ^,^ ^12^ Inflammatory cytokines and chemokines generated from this initial response may induce the proliferation of type 1 helper or type 2 helper T cells, which will promote cellular or humoral immune response, respectively.^13^ Although the aforementioned mechanisms are important clues to determine the evolution of the disease to the tuberculoid or lepromatous form, they are not sufficient to explain the leprosy spectrum.

Another innate immunity mechanism triggered by microbiological agents involves pattern recognition receptors (PRR), particularly the cytosolic NOD-like receptor (NLRs) group. The NLRP subfamily integrates cellular signaling complexes known as inflammasomes and triggers the maturation of pro-inflammatory molecules.^14^ ^,^ ^15^ The best characterised is the NLRP3 inflammasome, which contains the adapter protein apoptosis-associated speck-like protein (ASC) and procaspase-1.^16^ To ensure adequate and timely immune activity, the interactions among the NLRP3 inflammasome proteins regulate inflammasome function through three signaling pathways, all leading to increased interleukin-1β (IL-1β) and interleukin-18 (IL-18).^17^ ^,^ ^18^ In humans, the classic pathway requires caspase-1 activation, the pathway non-canonical requires caspases-4/5 while the alternative pathway requires caspase-8 for which only very limited evidence exists.^19^

Knowledge about activation of the inflammasome mediated pathway by mycobacteria remains limited and controversial. In the case of *Mycobacterium tuberculosis*, there are conflicting data as to whether or not this pathogen inhibits the activation of the inflammasome pathway.^20^ ^,^ ^21^ ^,^ ^22^ One study showed that infection with *M. leprae* reduced the activation of caspase-1 and IL-1β secretion in macrophages^23^ whereas another showed high levels of IL-1β in patients with leprosy regardless of the clinical form.^24^ More recently, one study indicated that expression of inflammasome markers in the lepromatous form of the infection with *M. leprae* points to the ineffectiveness of this protein complex in controlling the infection.^25^

In this study, we investigated the role of the NLRP3 inflammasome-mediated pathway in the immunopathological spectrum of leprosy by assessing the immunohistochemical expression of inflammatory biomarkers in skin lesions. Besides contributing to explain the role of the NLRP3 inflammasome in the different immunopathological forms of leprosy, our data suggest a possible mechanism of immune evasion of *M. leprae*.

## MATERIALS AND METHODS

*Study design* - This is an observational, cross-sectional, comparative study involving the immunohistochemical evaluation of 99 skin lesion samples obtained before treatment from patients diagnosed with leprosy and being treated at the outpatient Clinica de Dermatologia Osvaldo Costa of the Hospital das Clínicas of UFMG from 2000 to 2015. The average age of the participants was 51 years and all came from Minas Gerais, particularly the Metropolitan Region of Belo Horizonte and the Jequitinhonha and Mucuri valleys in Minas Gerais.

Considering the clinical and immunopathological aspects of leprosy, the following groups were defined for the study: LL/BL (lepromatous leprosy or lepromatous pole); TT/BT (tuberculoid leprosy or tuberculous pole); BB (non-polar forms lepromatous or tuberculoid leprosy); nsD (non-specific dermatitis, proven not to be associated or unrelated to leprosy).

We included samples larger than 3 mm in diameter derived from patients aged 18 - 75 years. Samples from immunocompromised, HIV/AIDS, tuberculosis, occurrence of leprosy reaction episodes and autoimmune diseases’ patients were excluded.

*Immunohistochemistry* - Histological slides containing 5 μm serial sections of the paraffin samples were incubated overnight in an oven at 56ºC. Subsequently, the samples were submitted to dewaxing and rehydration stages, with three washes in xylol for 5 min each, and three washes in ethyl alcohol (PA) for 5 min. After rehydration, heat-induced antigenic recovery was performed in 0.01 M sodium citrate solution (pH 6.0) at 90ºC for 20 min in the steam and cooled to room temperature for 20 min. Endogenous tissue peroxidase and nonspecific proteins were blocked at different stages according to the laboratory protocol.

The immunohistochemical labeling was done separately with the following monoclonal primary antibodies: anti-NLRP3 (Cryopyrin-H-66: sc-66846, Santa Cruz Biotechnology, INC), anti-Caspase-1 (Caspase-1-14F468: sc-56036, Santa Cruz Biotechnology, INC); anti-Caspase-11 (Caspase-11 p20-A2: sc-374615, Santa Cruz Biotechnology, INC); anti-IL-18 (IL-18-H173: sc-7954, Santa Cruz Biotechnology, INC);and anti-IL-1β (IL-1β-2H12: sc-130323, Santa Cruz Biotechnology, INC). Several tests, with skin samples donated by the Laboratório de Patologia Molecular (UFMG), were performed to standardise the primary antibodies. Dilutions of 1:500 were defined for primary anti-NLRP3, anti-Caspase-1, and anti-ILβ primary antibodies, and dilutions of 1:300 were defined for the primary anti-Caspase-11 and anti-IL-18 antibodies according to the manufacturer’s instructions.

The NovoLink Max Polymer Detection Novocastra™ kit (Leica Microsystems) was used to detect primary antibodies. The slices were incubated with the NovoLink kit universal polymer detection system for 30 min at room temperature. Subsequently, 200 μL of the developer solution supplied by the kit containing the chromogen diamino-benzidine 3,3 (DAB) was added and the reaction was incubated at room temperature for 5 min. Staining was performed by immersing the slides in Harris hematoxylin solution (Code 248, Vetec) for 30 s. The slides were analysed using an Olympus BX optical microscope. Qualitative and semi-quantitative analyses were carried out in random areas under 40x magnification by two independent observers and the final ranking was reached after they reached a consensus between them. To monitor the quality of the immunohistochemical reactions, external and internal controls were used. The external control involved five healthy skin samples obtained from breast biopsies whereas the internal controls analysed the epithelial compartment of the study samples. The NLRP inflammasomes have been shown to be expressed in normal human epidermal keratinocytes.^26^

We analysed the intensity and the percentage of cellular immunoreactivity of immunohistochemical reactions of the inflammatory infiltrate. Subsequently, a score of immunohistochemical expression was established, based on a previously validated score.^11^ The product of the variables intensity and percentage was ranked as overexpressed if ≥ 4. So we compared the biomarkers immunohistochemical expression between the groups by calculating high/ strong and low scores.

*Statistical analyses* - The results were stored in spreadsheets using the EXCEL program and analysed with the R (version 3.1.2) and MINITAB 17 (version 17) programs. For descriptive analyses, the categorical variables of the qualitative and semi-quantitative approaches were presented as numbers and percentages. Student’s t-test was used to compare difference between groups. To establish a relationship between immunohistochemical expression scores and the study groups, a multinomial logistic regression model was fitted using the logistic function in the R and VGAM package. Statistically significant differences were considered when p < 0.05.

*Ethics* - The present study followed all guidelines for research involving human beings, displayed in the Resolution 466/2012 of the Brazilian National Health Council to safeguard the rights and well-being of study participants. This research was approved by the Research Ethics Committee (COEP) of the Universidade Federal de Minas Gerais (UFMG) under the CAAE no. 14887414.0.0000.5149.

## RESULTS

**Fig. 1:**
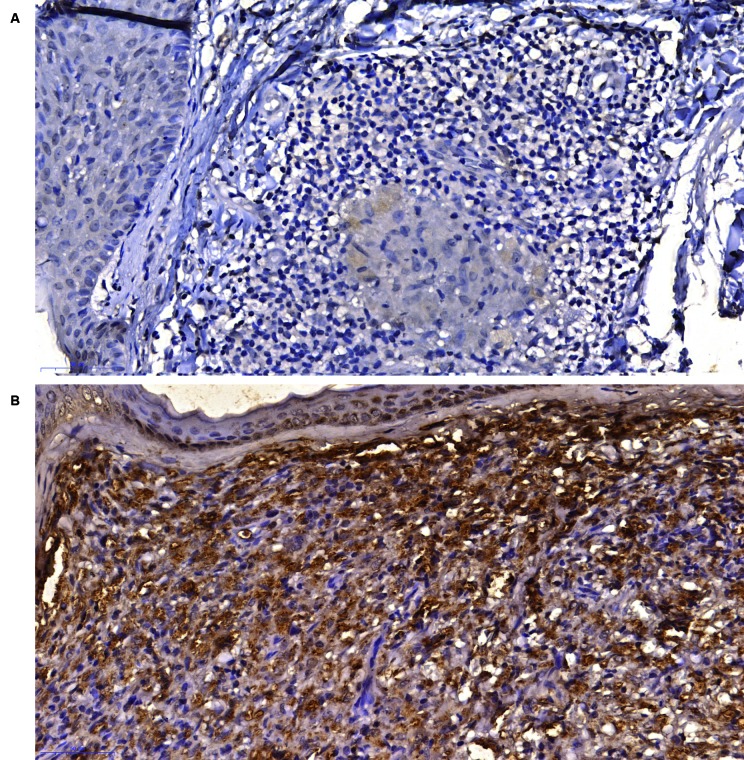
expression of NLRP3 in the immunopathological forms of leprosy. (A) polar tuberculoid (TT)/borderline tuberculoid (BT)-group showing mild NLRP3 staining in granuloma macrophages (400x magnification; Bar: 50 µM); (B) lepromatous leprosy (LL)/ borderline lepromatous (BL) group showing intense and diffuse staining for NLRP3 in macrophages (400x magnification; Bar: 50 µM).

*NLRP3 is overexpressed in lepromatous leprosy* - The immunohistochemical data presented in Fig. 1 shows that samples from LL/BL had intense or moderate intensity of NLRP3. In the other spectrum groups, immunoreactivity with light intensity predominated. Considering the inflammatory infiltrate, approximately 95% of the samples from the LL/BL group and 80% of the BB group displayed moderate or intense NLRP3 immunoreactivity. Most of the samples from the LL/BL group presented reactivity in more than 50% of the inflammatory cells.

**Fig. 2:**
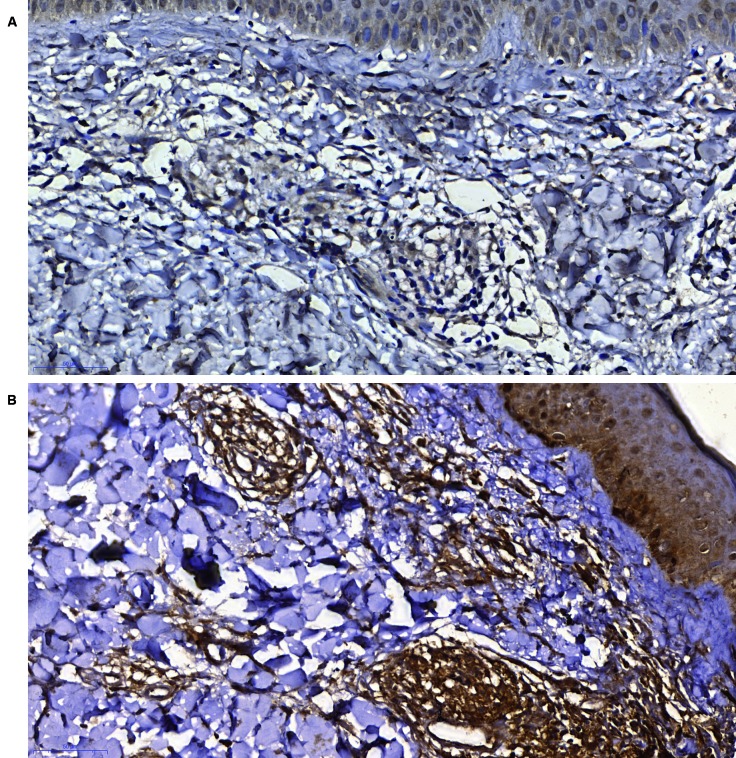
expression of caspases-4/5 in the immunopathological forms of leprosy. (A) polar tuberculoid (TT)/ borderline tuberculoid (BT)-group showing slight staining for caspases-4/5 in inflammatory cells (400x magnification; Bar: 50 µM); (B) borderline borderline (BB)-group presenting intense staining for caspases-4/5 in inflammatory cells (400x magnification; Bar: 50 µM).

Fig. 2 shows that caspases-4/5 only presented moderate or intense intensity in the inflammatory infiltrate of the LL/BL group. In the other spectrum groups, immunoreactivity intensity was mostly light or absent. We observed that more than 50% of inflammatory cells showed reactivity in 60% of the LL/BL samples. All samples from the other spectrum groups showed a percentage of cellular immunoreactivity smaller than 25%.

Immunoreactivities of caspase-1, IL-1β, and IL-18 were absent in the inflammatory infiltrate in all groups.

*NLRP3 inflammasome’s immunohistochemical strong expression score is a hallmark of the Lepromatous Leprosy* - The immunohistochemical expression of NLRP3, caspase-1, caspases-4/5, IL-1β, IL-6, and IL-18 between the groups presented statistically significant differences in the scores (Table I). The data reveal significant differences between the LL/BL and the nsD and TT/BT groups for all biomarkers (p = 0.000). Notably, the expression ofNLRP3 in the LL/BL group scored mostly strong. In contrast, the other groups scored low and the difference between them and the LL/BL group was statistically significant (p = 0.000). Expression of caspases-4/5 in the LL/BL group was split between low and strong scores and statistically differed from the other spectrum groups, which presented mostly low scores (p = 0.000). Finally, all groups presented low scores for caspase-1, IL-1β, and IL-18 expression in the inflammatory infiltrate.

**TABLE I:** Immunohistochemical scores of NLRP3, caspase-1, caspases-4/5, IL-1β, IL-6, and IL-18 expression in the inflammatory infiltrate compartment

Biomarker	Score	Group							
		nsD		TT/BT		BB		LL/BL	
		n	(%)	n	(%)	n	(%)	n	(%)
NLRP3	0	17	(100.00)	26	(96.30)	8	(72.73)	4	(10.26)
	1	0	(0.00)	1	(3.70)	3	(27.27)	35	(89.74)
Caspases-4/5	0	18	(100.00)	25	(92.59)	9	(81.82)	18	(46.15)
	1	0	(0.00)	2	(7.41)	2	(18.18)	21	(53.85)
Caspase-1	0	17	(100.00)	26	(96.30)	10	(100.00)	33	(91.67)
	1	0	(0.00)	1	(3.70)	0	(0.00)	3	(8.33)
IL-1β	0	17	(100.00)	28	(100.00)	9	(100.00)	31	(100.00)
	1	0	(0.00)	0	(0.00)	0	(0.00)	0	(0.00)
IL-18	0	17	(100.00)	28	(100.00)	11	(100.00)	24	(85.70)
	1	0	(0.00)	0	(0.00)	0	(0.00)	4	(14.30)

BB: borderline borderline; BL: borderline lepromatous; BT: borderline tuberculoid; LL: lepromatous leprosy; nsD: non-specific dermatitis; TT: polar tuberculoid.

**TABLE II:** Estimated coefficients, standard errors (SE), z-scores, two-tailed p-values, and odds ratio (OR) for the final fitted multinomial logistic regression model

Predictor	Coefficients	SE	z-score	p value	OR	95%CI
Logit 1: (LL/BL / TT/BT)					227.50	(24.00;2156.94)
Constant	-1.87	0.53	-3.49	0.000*		
NLRP3	5.42	1.14	4.73	0.000*		
Logit 2: (nsD / TT/BT)					0.00	**
Constant	-0.42	0.31	-1.36	0.173		
NLRP3	-0.18	6500.13	-0.00	0.998		
Logit 3: (BB / TT/BT)					9.75	(9.75;107.25)
Constant	-1.17	0.40	-2.92	0.004*		
NLRP3	2.27	1.22	1.86	0.03		
Log-likelihood: -80,493						

*: denotes statistically significant differences (p < 0.05). **: no goodness-of-fit test performed.

*NLRP3 overexpression is associated with high risk of lepromatous leprosy* - Table II summarises the results of the relationship between leprosy spectrum groups and NLRP3 inflammasome expression. According to the model, only the coefficient NLRP3 in Logit 1 (LL/BL / TT/BT) presented statistical significance. However, Logit 3 (BB / TT/BT), indicates a trend for statistical significance and a high possibility of adjustment. The model deviance residuals indicated a general goodness-of-fit of the model to the obtained data.

Table II also presents the odds ratio (OR) values for the predictors, with confidence intervals (95% CI) calculated from the final model. The results indicate substantial increases in the odds of NLRP3 overexpression in the LL/BL and BB groups in comparison with the TT/BT group, suggesting a predictive role for NLRP3.

## DISCUSSION

The present study presents relevant and original results regarding the participation of the NLRP3 inflammasome in the immunopathogenesis of leprosy. We found overexpression of NLRP3 and caspases-4/5 biomarkers in the multibacillary forms (LL/BL and BB), particularly in the lepromatous form of leprosy. Interestingly, the expression pattern of these biomarkers in the multibacillary forms was similar in skin tissue analysed, albeit the frequency of cells overexpressing these biomarkers was higher in the LL/BL than in the BB group. We also observed poor expression of caspase-1, IL-1β, and IL-18 in all leprosy spectrum groups.

Recent evidence suggests the involvement of the NLRP3 inflammasome in the inflammatory response induced by caspase-11 in response to bacterial infections that affect the cytoplasm of host cells such as *M. tuberculosis* and *M. leprae*.^19^ ^,^ ^25^ However, the innate stimuli that activate these pathways remain unknown and corroborating a previous study, our results show that there is probably ineffectiveness of this protein complex in controlling the infection in lepromatous lesions.^25^

We speculate that the strong expression of the NLRP3 and caspases-4/5 in the samples of multibacillary forms may be due to the high concentration of *M. leprae* bacilli in these tissues. Also, it is possible that the absence or the small number of viable bacilli present in the lesions of the tuberculoid pole is not sufficient to activate the inflammasome, which would explain why NLRP3 and caspases-4/5 are poorly expressed in these samples. Future research will be needed to explore this hypothesis.

The activation of the non-canonical pathway NLRP3 observed in the multibacillary form of leprosy may occur through a mechanism similar to that observed in response to Gram-negative bacteria such as *Escherichia coli*, *Citrobacter rodentium*, and *Vibrio cholerae*. The external membrane of these bacteria is constituted mainly of LPS molecules that bind and directly activate caspase-11, an orthologue of human caspases-4/5.^19^ However, it is still unknown which component(s) of *M. leprae* could be responsible for the activation of this pathway. Interestingly, *M. tuberculosis* inhibits NLRP3 inflammasome activation to block the processing of caspase-1 and IL-1β,^21^ a finding that matches the observations for the tuberculoid pole described herein.

The first step of the classical pathway of NLRP3 inflammasome activation involves the recognition pathogen-associated molecular patterns (PAMPS) or danger-associated molecular patterns (DAMPS) by TLRs, leading to activation of nuclear factor kappa B (NF-κB)-mediated signaling, which in turn up-regulates transcription of inactive NLRP3, pro-IL-1β, and pro-IL-18. The second step is the oligomerisation of NLRP3 following by the assembly of NLRP3, ASC, and pro-caspase-1 into a complex. This triggers the self-activation of pro-caspase-1 into the enzymatically active by proteolytic cleavage, as well as the production and secretion of mature IL-1β and IL-18.^17^ Most of the studies about the non-canonical pathway of NLRP3 inflammasome activation have been performed with Gram-negative bacteria. It requires the previous activation of caspase-11 and human caspases-4/5 by microbial products or particles such as LPS.^19^ However, the signaling mechanism upstream of caspase-11 activation remains controversial.

In the dermal microenvironment of samples of the tuberculoid pole (TT/BT group), a type 1 adaptive response is triggered by lymphocytes, histiocytes, monocytes, dendritic cells and endothelium, as well as keratinocytes and fibroblasts.^7^ ^,^ ^26^ This response is characterised by a high expression of IFN-γ and IFN-β stimulated by the presence of *M. leprae* fragments such as DNA residues.^7^ ^,^ ^13^ We suggest that the increased expression of IFN- β and IFN-γ may induce the transcription of NLRP3, pro-IL-1β, and pro-IL-18 and, consequently, oppose the activation of the NLRP3 inflammasome. This mechanism does not depend on the inflammatory response elicited by dermatitis of non-specific etiology (group nsD).

In lepromatous leprosy (LL/BL group), there is a large presence of *M. leprae* bacilli in the macrophages and a dermal microenvironment with type 2 response pattern, with low expression of interferons, mainly IFN-γ. In this case, the large number of bacilli may be the main stimulatory factor leading to the transcription of NLRP3 and caspases-4/5. However, the expression of the caspase-1, IL-1β, and IL-18 is absent in the lepromatous form because of possible effects on the transcription and/or activation of caspase-1. Indeed, our immunohistochemical expression results observed in the BB and LL/BL groups suggest the inactivation or non-participation of the NLRP3 inflammasome and the immune evasion of *M. leprae* in this form of leprosy.

The study’s main limitation is the risk of subjective interpretation of immunohistochemistry results. To mitigate this risk three precautions were taken: (i) qualitative or descriptive analysis of immunohistochemical reactions by two independent variables (intensity and percentage of reactivity); (ii) semi-quantitative analysis of immunohistochemical reactions at the same sites using a validated immunohistochemical expression; and (iii) classification of immunohistochemical reactions by a consensus between two independent observers.

The results reported here in allow us to conclude that, despite the overexpression of NLRP3 and caspases-4/5 the lepromatous pole, the NLRP3 inflammasome does not actively participate in the innate immune response in leprosy. Together, these results may help better understand the prediction of the clinical evolution of leprosy and the search for biomarkers for morbidity, prognosis, and therapeutic response of this neglected disease.

## References

[1] WHO - World Health Organization (2012). Leprosy: fact sheet no. 101.

[2] Ridley DS, Jopling WH (1966). Classification of leprosy according to immunity A five-group
system. Int J Lepr Other Mycobact Dis.

[3] Eichelmann K, Gonzalez Gonzalez SE, Salas-Alanis JC, Ocampo-Candiani J (2013). Leprosy An update: definition, pathogenesis, classification,
diagnosis, and treatment. Actas Dermosifiliogr.

[4] Alter A, Grant A, Abel L, Alcaïs A, Schurr E (2011). Leprosy as a genetic disease. Mamm Genome.

[5] Ottenhoff TH (1994). Immunology of leprosy New developments. Trop Geogr Med.

[6] Jacobson RR, Krahenbuhl JL (1999). Leprosy. Lancet.

[7] Fonseca AB, Simon MD, Cazzaniga RA, Moura TR, Almeida RP, Duthie MS (2017). The influence of innate and adaptative immune responses on the
differential clinical outcomes of leprosy. Infect Dis Poverty.

[8] Gomes GI, Nahn EP, Santos RK, da Silva WD, Kipnis TL (2008). The functional state of the complement system in
leprosy. Am J Trop Med Hyg.

[9] Schlesinger LS, Horwitz MA (1991). Phenolic glycolipid-1 of Mycobacterium leprae binds complement
component C3 in serum and mediates phagocytosis by human
monocytes. J Exp Med.

[10] Krutzik SR, Ochoa MT, Sieling PA, Uematsu S, Ng WY, Legaspi A (2003). Activation and regulation of Toll-like receptors 2 and 1 in human
leprosy. Nat Med.

[11] Oliveira RB, Ochoa MT, Sieling PA, Rea TH, Rambukkana A, Sarno EN (2003). Expression of Toll-like receptor 2 on human Schwann cells a
mechanism of nerve damage in leprosy. Infect Immun.

[12] Bochud PY, Hawn TR, Aderem A (2003). Cutting edge a Toll-like receptor 2 polymorphism that is
associated with lepromatous leprosy is unable to mediate mycobacterial
signaling. J Immunol.

[13] Manca C, Peixoto B, Malaga W, Guilhot C, Kaplan G (2012). Modulation of the cytokine response in human monocytes by
mycobacterium leprae phenolic glycolipid-1. J Interferon Cytokine Res.

[14] Latz E, Xiao TS, Stutz A (2013). Activation and regulation of the inflammasomes. Nat Rev Immunol.

[15] Schroder K, Tschopp J (2010). The inflammasomes. Cell.

[16] He Y, Hara H, Nunez G (2016). Mechanism and regulation of NLRP3 inflammasome
activation. Trends Biochem Sci.

[17] Jin C, Flavell RA (2010). Molecular mechanism of NLRP3 inflammasome
activation. J Clin Immunol.

[18] Yang J, Liu Z, Xiao TS (2017). Post-translational regulation of inflammasomes. Cell Mol Immunol.

[19] Stowe I, Lee B, Kayagaki N (2015). Caspase-11 arming the guards against bacterial
infection. Immunol Rev.

[20] Wong KW, Jacobs WR (2011). Critical role for NLRP3 in necrotic death triggered by
Mycobacterium tuberculosis. Cell Microbiol.

[21] Master SS, Rampini SK, Davis AS, Keller C, Ehlers S, Springer B (2008). Mycobacterium tuberculosis prevents inflammasome
activation. Cell Host Microbe.

[22] Briken V, Ahlbrand SE, Shah S (2013). Mycobacterium tuberculosis and the host cell inflammasome a
complex relationship. Front Cell Infect Microbiol.

[23] Kang TJ, Lee GS, Kim SK, Jin SH, Chae GT (2010). Comparison of two mice strains, A/J and C57BL/6, in caspase-1
activity and IL-1beta secretion of macrophage to Mycobacterium leprae
infection. Mediators Inflamm.

[24] Costa RD, Mendonca VA, Lyon S, Penido RA, Costa AMDD, Costa MD (2008). Evaluation of the expression of interleukin 1 beta (IL-1beta) and
interleukin 1 receptor antagonist (IL-1Ra) in leprosy
patients. Rev Soc Bras Med Trop.

[25] Silva LM, de Sousa JR, Hirai KE, Dias LB, Furlaneto IP, Carneiro FRO (2018). The inflammasome in leprosy skin lesions an immunohistochemical
evaluation. Infect Drug Resist.

[26] Gruber JV, Holtz R (2019). In vitro expression of NLRP inflammasome-induced active Caspase-1
expression in normal human epidermal keratinocytes (NHEK) by various
exogenous threats and subsequent inhibition by naturally derived ingredient
blends. J Inflamm Res.

